# Relationship between decision regret, anxiety, and depression in surrogate decision-makers for neurocritical patients with prolonged disorders of consciousness: a cross-lagged analysis

**DOI:** 10.1186/s40359-026-04870-2

**Published:** 2026-05-25

**Authors:** Xinyu Zhang, Sailu Mao, Sihua Wang, Jia Yu, Rongqing Li, Zikai Zhang, Yang Chen, Li Zeng

**Affiliations:** 1 Nursing Department, Tongji Hospital, School of Medicine, Tongji University, Shanghai, 200065 China; 2 Nursing Department, Shanghai Tenth People’s Hospital, School of Medicine, Tongji University, Shanghai, 200072 China; 3 Department of Cardiothoracic Surgery, Tongji Hospital, School of Medicine, Tongji University, Shanghai, 200065 China; 4 Department of Science Administration, Tongji Hospital, School of Medicine, Tongji University, Shanghai, 200065 China

**Keywords:** Critically Ill Neurological Patients, Disorders of Consciousness, Surrogate Decision-Makers, Decision Regret, Anxiety, Depression, Cross-Lagged Analysis

## Abstract

**Background:**

Decision regret is common among surrogate decision-makers for neurocritical patients with prolonged disorders of consciousness (pDoC). This regret, which stems from uncertain patient preferences and complex critical care decisions, predisposes surrogates to adverse psychological outcomes, including anxiety and depression. However, as most evidence on this association comes from cross-sectional studies, the directional relationships and underlying mechanisms between decision regret and psychological distress remain unclear. This prospective longitudinal study employed a cross-lagged panel analysis to delineate the longitudinal trajectory of these symptoms and to explore their bidirectional relationships.

**Methods:**

We conducted a prospective longitudinal study from January to November 2024, recruiting 227 surrogate decision-makers for neurocritical patients with pDoC from two tertiary hospitals in Shanghai, China. We assessed decision regret, anxiety, and depression using the Decision Regret Scale and the Hospital Anxiety and Depression Scale at one (T1), three (T2), and six (T3) months after the patient’s diagnosis. Cross-lagged panel models examined the bidirectional relationships between heightened decision regret and symptoms of anxiety and depression.

**Results:**

The average level of decision regret among surrogate decision-makers initially peaked and subsequently declined over time. In contrast, symptoms of anxiety and depression demonstrated a gradual decrease. The incidence rates of heightened decision regret at T1, T2, and T3 were 23.18% (*n* = 54), 34.33% (*n* = 80), and 18.03% (*n* = 42), respectively. Cross-lagged analyses revealed a bidirectional relationship between heightened decision regret and anxiety from T1 to T2 (*β* = 0.327, *p* < 0.05; *β* = 0.113, *p* < 0.001). From T2 to T3, however, only anxiety predicted subsequent heightened decision regret (*β* = 0.052, *p* < 0.05). A bidirectional relationship was also observed between heightened decision regret and depression from T1 to T2 (*β* = 0.321, *p* < 0.05; *β* = 0.094, *p* < 0.05).

**Conclusions:**

This study found a reciprocal relationship and time‑dynamic relationship between heightened decision regret and symptoms of anxiety and depression of in surrogate decision-makers for neurocritical patients with pDoC. Specifically, from T1 to T2, heightened decision regret had bidirectional relationships with anxiety and with depression, respectively; from T2 to T3, only anxiety predicted subsequent heightened decision regret. Therefore, clinical staff should dynamically assess the psychological status of surrogate decision-makers in a stage-specific manner and implement differentiated intervention strategies accordingly.

**Trial registration number:**

Not applicable.

## Introduction

Neurological critically ill patients often present with severe disorders of consciousness, which are characterized by varying degrees of impairment in perceiving and responding to internal and external stimuli [1]. A duration exceeding 28 days defines a prolonged disorder of consciousness (pDoC) [2], with an estimated 100,000–300,000 cases in the United States and a European prevalence of 0.2–6.1 per 100,000 people [3]. Owing to the nature of their condition and necessary therapeutic interventions, patients with pDoC in neurocritical care settings typically lose decision-making capacity. Consequently, surrogate decision-makers must assume responsibility for making medical choices on their behalf. Approximately 95% of critically ill patients in the intensive care unit (ICU) rely on surrogate decision-makers to participate in complex medical decisions regarding life-support interventions, such as mechanical ventilation and cardiopulmonary resuscitation [4].

Factors such as unknown patient preferences, complex critical care choices, and insufficient support contribute to a negative decision-making experience, frequently resulting in decision regret [5]. Studies indicate that the incidence of decision regret among surrogates in adult ICUs can be as high as 73% [6]. Decision regret is defined as a negative cognitive-emotional response following a healthcare decision, manifesting as persistent rumination and negative emotions [7]. This emotional response impairs the decision-making process by reducing surrogate efficacy and increasing cognitive load, which can result in delays or compromised decision quality that ultimately affect patient outcomes [8]. Research indicates that surrogate decision-makers experience sustained high psychological burden over extended periods, and behind this phenomenon exist deeper psychological mechanisms [9]. According to Boss’s theory of ambiguous loss, when a loved one is physically present but psychologically absent (e.g., in a state of disorder of consciousness) [10, 11], individuals enter a state of grief that lacks clear closure. For surrogate decision‑makers of pDoc patients, they often find it difficult to accept the established medical outcome, tend to ruminate on and doubt past choices, thereby inducing decision regret, which directly exacerbates their anxiety and depression. Therefore, investigating the predictors of decision regret among surrogates of neurocritical patients with pDoC is essential for developing interventions to mitigate regret, improve the decision-making experience, and enhance decision quality.

The intense stress of the ICU environment, combined with critical patient illness, prognostic uncertainty, and substantial financial burden, exposes surrogate decision-makers to profound psychological distress, which frequently manifests as anxiety and depression [12]. These common negative emotions can also have lasting effects on their mental health. Studies report a high prevalence of psychological distress among surrogate decision-makers, with anxiety affecting 15% to 94.2% and depression affecting 6.6% to 70% [13]. Such distress often begins at the time of the patient’s ICU admission and may persist for months after transfer or death, substantially impairing the surrogate’s own health and quality of life [14]. Furthermore, anxiety and depressive symptoms can compromise cognitive function and decision-making capacity in surrogate decision-makers, thereby reducing decision quality and increasing vulnerability to decision regret [15].

Existing evidence suggests that decision regret may worsen symptoms of anxiety and depression in this population. Conversely, pre-existing anxiety and depression may influence the decision-making process and subsequently intensify regret. Thus, a reciprocal relationship may exist between decision regret and symptoms of anxiety and depression among surrogate decision-makers. However, most studies on this topic are cross-sectional and have focused primarily on whether anxiety and depression serve as influential factors for decision regret. In addition, decision regret exhibits temporal variation, demonstrating a dynamic trajectory over time [16]. In longitudinal research, it remains unclear how levels of decision regret among surrogate decision-makers for neurocritical patients with pDoC evolve over time, and what dynamic interrelationships exist between this regret and symptoms of anxiety and depression.

Therefore, this study employed a prospective longitudinal design and cross-lagged panel model to examine the reciprocal relationship between decision regret and symptoms of anxiety and depression among surrogate decision-makers of neurocritical patients with pDoC. The specific aims were to investigate: (a) whether decision regret unidirectionally predicts anxiety, anxiety unidirectionally predicts regret, or a bidirectional relationship exists between them; and (b) whether decision regret unidirectionally predicts depressed mood, depressed mood unidirectionally predicts regret, or a bidirectional relationship exists between them.

## Methods

### Participants and procedures

#### Participants

This prospective longitudinal study, conducted from January to November 2024, investigated surrogate decision-makers for neurocritical patients with pDoC. A convenience sample was recruited from the neurosurgical and neurological ICUs of two tertiary hospitals in Shanghai, China.

Patient inclusion criteria were: (1) diagnosis consistent with neurocritical illness as defined by the *Expert Consensus on Management of Neurocritical Illnesses* [17], which mainly includes cerebrovascular diseases, traumatic brain injury, brain tumors, among others; (2) duration of impaired consciousness ≥ 28 days, Glasgow Coma Scale (GCS) score < 12, stable vital signs, and availability of complete clinical data. According to the GCS grading standard [18], a GCS score of 9–12 is defined as moderate disorder of consciousness, and a score of 3–8 is defined as severe disorder of consciousness or coma. Therefore, all patients included in this study were at the level of moderate to severe disorder of consciousness.

Inclusion criteria for surrogate decision-makers were: (1) age ≥ 18 years and cognitively intact; (2) designation as the primary caregiver with decisional authority for the patient’s daily care and medical treatment; (3) adequate communication and comprehension abilities; and (4) provision of informed consent and voluntary agreement to participate.

**Fig. 1:**
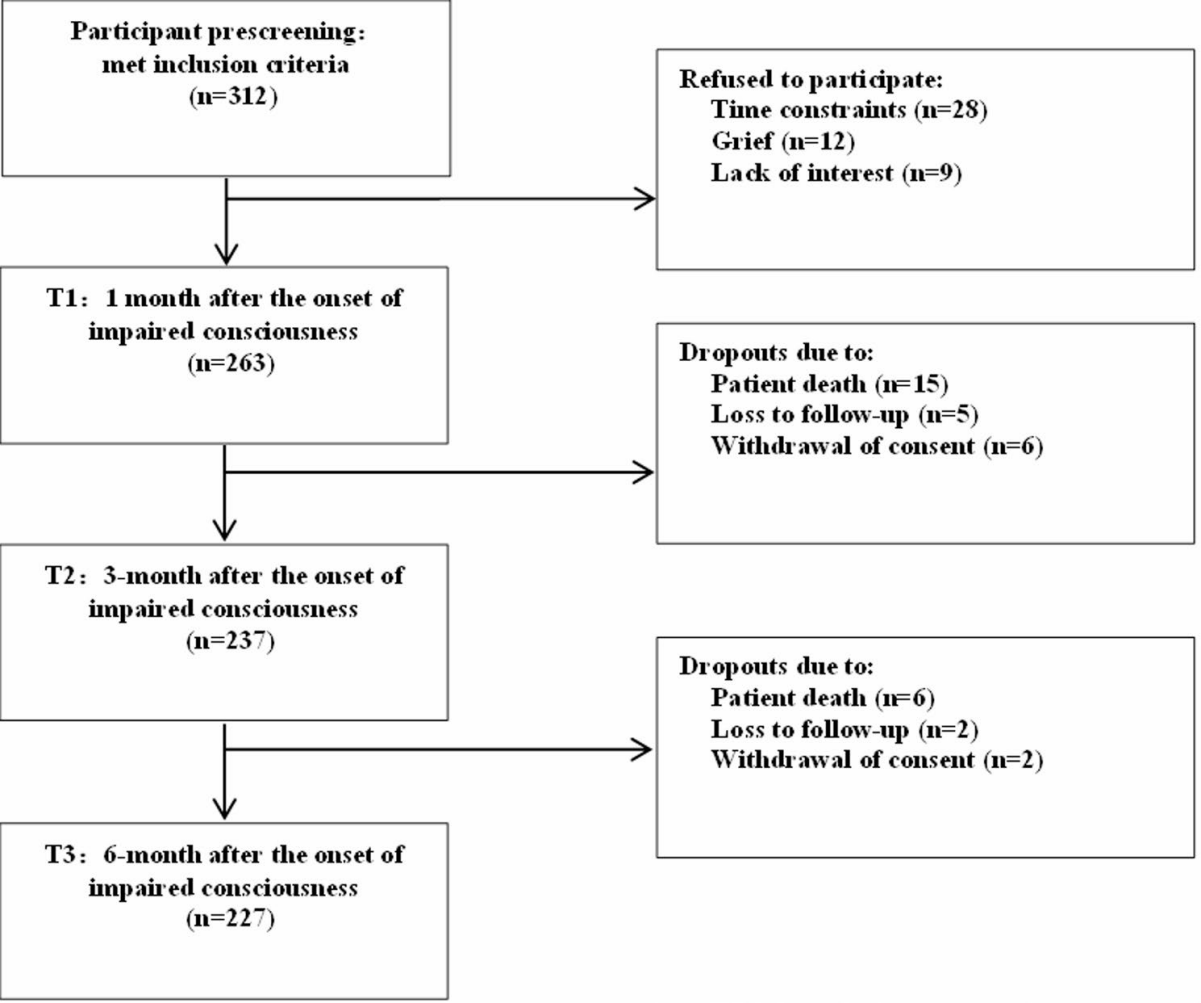
Flow diagram of sample selection

During the study period, a total of 312 patients met the inclusion criteria. Among them, the surrogate decision-makers of 263 patients were successfully contacted and agreed to participate in the study. The main reasons for refusal among the remaining 49 decision-makers were lack of time (*n* = 28), being too overwhelmed by grief to accept the survey (*n* = 12), and lack of interest (*n* = 9). Ultimately, 227 surrogate decision-makers completed all follow-up assessments. The attrition rates were 9.9% at T2 and 3.8% at T3, resulting in an overall attrition rate of 13.7% throughout the study. Participant flow is detailed in Fig. 1.

#### Procedures

The progression of disorders of consciousness in neurocritical patients follows a staged trajectory. When impaired consciousness persists beyond 28 days, the condition transitions to a pDoC. The subsequent month represents the subacute stage, a pivotal window for clinical progression. By three months, neurological recovery typically reaches a plateau phase, characterized by significantly slowed improvement as function stabilizes or deteriorates. Beyond six months, the condition is widely considered to have entered the chronic or sequela stage, marked by established neurological deficits and a necessary shift toward long-term complication management. Accordingly, levels of decision regret among surrogate decision-makers were assessed at three time points following the onset of impaired consciousness: one month (T1), three months (T2), and six months (T3).

In this study, three trained specialized nurses performed daily screening of patients in the neurosurgical and neurological ICUs across participating hospitals. Once a patient met the neurocritical diagnosis criteria, with a GCS score > 12 and impaired consciousness persisting beyond 28 days, the specialized nurse verified the inclusion and exclusion criteria for both the patient and the surrogate decision‑maker. For patients who met the criteria, the specialized nurse provided a detailed explanation of the study purpose and procedures to the surrogate decision‑maker, and the patient was enrolled after written informed consent was obtained. Specialized nurses completed enrollment registration and T1 paper‑based data collection during hospitalization. For T2 and T3, when patients might have already been discharged, the nurses established a WeChat follow‑up group before discharge, conducted regular one‑to‑one follow‑ups, and collected data via telephone or paper questionnaires at outpatient visits. In accordance with the principles of the Declaration of Helsinki, this study was approved by the Ethics Committee of Shanghai Tongji Hospital (approval number: K-2024-019). Participants were assured that their responses would remain strictly anonymous and confidential, and that they could withdraw from the study at any time.

### Measurement

#### Demographics

A socio-demographic questionnaire was developed based on a comprehensive literature review. This instrument collected patient characteristics, including age, gender, insurance type, average monthly household income per capita, diagnosis, length of ICU stay, APACHE II score, and the presence of an advance care plan. For surrogate decision-makers, the collected data encompassed age, gender, relationship to the patient, educational level, employment status, religious affiliation, prior ICU care experience, and their specific role in the decision-making process. Decision-making engagement was self-reported by surrogate decision-makers based on their actual experience in the medical decision-making process. It was classified into three categories: passive participation (decisions made primarily by the physician), collaborative participation (decisions made after discussion with the physician and family members), and active participation (decisions made independently by the surrogate decision-maker).

#### Decision regret

Decision regret was assessed using the Decision Regret Scale (DRS). The original 5-item DRS was developed by Brehaut in 2003 to measure regret following healthcare decisions and has since become the most widely used instrument for this purpose [19]. Items are rated on a 5-point Likert scale ranging from “strongly agree” to “strongly disagree” (scores 1–5). Items 2 and 4 are reverse-scored. The total score is converted to a percentage, with higher scores indicating greater regret; a score above 25 indicates a high level of decision regret. The scale demonstrates good internal consistency, with reported Cronbach’s α values ranging from 0.81 to 0.92. In 2019, Haun and colleagues adapted the original scale via semantic reframing for the caregiver population, resulting in the Caregiver Decision Regret Scale, which is designed to measure regret among surrogate decision-makers [20]. This adapted 5-item instrument retains the original scoring rules and has shown good internal consistency, with a reported Cronbach’s α of 0.83. The original Decision Regret Scale was adopted in this study to evaluate decision regret, as it is a more mature instrument and has been extensively applied and validated across various medical decision‑making scenarios.

#### Anxiety and depression

Anxiety and depressive symptoms in surrogate decision-makers were assessed using the Hospital Anxiety and Depression Scale (HADS), developed by Zigmond [21]. The HADS consists of two 7-item subscales measuring anxiety (HADS-A) and depression (HADS-D). Each item is scored from 0 to 3, with subscale totals ranging from 0 to 21. A score of ≥ 8 on either subscale indicates clinically significant symptoms, with higher scores reflecting greater severity. The scale demonstrated excellent reliability in this sample, with Cronbach’s α coefficients of 0.890 for the full scale, 0.820 for HADS-A, and 0.807 for HADS-D. In this study, a score of ≥ 8 was defined as indicating clinically significant anxiety or depressive symptoms.

### Data analysis

Data were analyzed using SPSS 27.0 and Mplus 8.3. Descriptive statistics summarized general participant characteristics, decision regret, and anxiety-depression scores. Pearson correlations examined relationships among these variables. Changes over time were assessed using repeated-measures ANOVA. To facilitate clinical identification of high‑risk individuals, we converted decision regret into a dichotomous variable based on the cutoff value recommended by the scale developers and performed cross‑lagged analyses. Two cross‑lagged panel models were established using Mplus 8.3. In this study, we operationalized decision regret in two ways: (1) as a continuous variable (raw score) for descriptive analyses (repeated-measures ANOVA), correlation analyses; (2) as a dichotomous variable indicating heightened decision regret (defined as a score > 25 points), which was used for the cross-lagged panel analysis.

Given that CLPM is designed to examine reciprocal influences among key psychological variables rather than adjusting for external covariates [22], and because the relevant demographic and clinical variables showed no significant association with heightened decision regret in this study (all *p* > 0.05), we did not include any covariates in the final model. Additionally, given the limited sample size, adding multiple covariates would have substantially increased the number of parameters to be estimated, risking model instability and reduced statistical power. Therefore, based on both theoretical and statistical considerations, the final model excluded covariates.

Model fit was evaluated using the following criteria [23]: Comparative Fit Index (CFI) ≥ 0.90, Tucker-Lewis Index (TLI) ≥ 0.90, Root Mean Square Error of Approximation (RMSEA) < 0.08, and Standardized Root Mean Square Residual (SRMR) < 0.08. Missing data were handled using the Robust Maximum Likelihood estimator, which is appropriate for longitudinal designs with attrition. A two-tailed *p* < 0.05 defined statistical significance for all tests.

## Results

### Descriptive statistics and correlation analysis

**Fig. 2:**
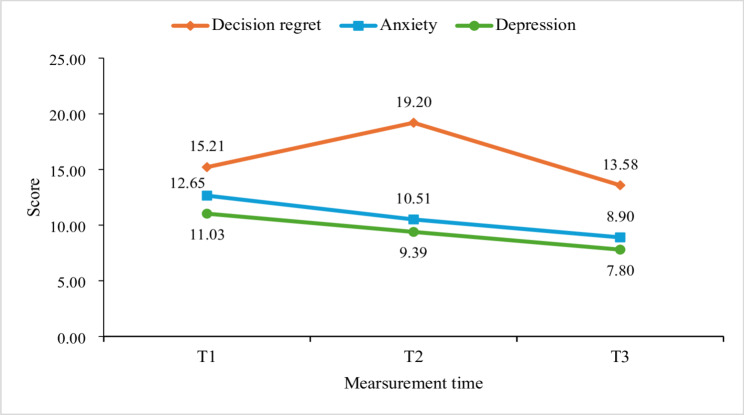
The score of decision regret, anxiety and depression among surrogate decision-makers. Note: Decision regret is presented as a continuous variable. T1, 1 month after the onset of impaired consciousness; T2, 3 months after onset; T3, 6 months after onset. Decision regret was measured using the Decision Regret Scale (DRS). Anxiety and depression were assessed using the Hospital Anxiety and Depression Scale (HADS)

The general characteristics of neurocritical patients with pDoC and their surrogate decision-makers are summarized in Table 1. Means and standard deviations for all study variables at each time point are provided in Fig. 2; Table 2. The incidence of decision regret among surrogate decision-makers was 52.36% (*n* = 122) at T1, 45.92% (*n* = 107) at T2, and 36.48% (*n* = 85) at T3. The incidence of heightened decision regret was 23.18% (*n* = 54) at T1, 34.33% (*n* = 80) at T2, and 18.03% (*n* = 42) at T3. Regarding psychological symptoms, the incidence of anxiety among surrogates was 94.42% (*n* = 220) at T1, 72.10% (*n* = 168) at T2, and 56.22% (*n* = 131) at T3. The incidence of depressive symptoms was 86.70% (*n* = 202) at T1, 62.23% (*n* = 145) at T2, and 39.91% (*n* = 93) at T3.

**Table 1:** Descriptive characteristics of the sample

		*N* (%)	M (SD)
Patients			
Gender			
	Female	71 (31.28)	
	Male	156 (68.72)	
Insurance type			
	Urban employee/resident basic medical insurance	138 (60.79)	
	New rural cooperative medical insurance	87 (38.33)	
	Others	2 (0.88)	
Monthly household income per capita (CNY)			
	< 5000	69 (30.40)	
	5000 ~ 10,000	103 (45.37)	
	> 10,000	55 (24.23)	
Disease type			
	Cerebrovascular disease	144 (63.44)	
	Traumatic brain injury	72 (31.72)	
	Brain tumor	11 (4.84)	
Advance care planning			
	No	223 (98.24)	
	Yes	4 (1.76)	
Age (years)			57.15 (17.20)
APACHE II score			20.83 (5.23)
ICU length of stay			30.19 (23.35)
Surrogate decision-makers			
Age (years)			46.71 (10.46)
Gender			
	Female	117 (51.54)	
	Male	110 (48.46)	
Education			
	Junior high school and lower	95 (41.85)	
	High school or technical secondary school	72 (31.72)	
	Undergraduate and above	60 (26.43)	
Religious affiliation			
	No	203 (89.43)	
	Yes	24 (10.57)	
Relationship to the patient			
	Parents	31 (13.66)	
	Adult child	106 (46.70)	
	Spouse	82 (36.12)	
	Sibling	8 (3.52)	
Employment status			
	Unemployed	40 (17.62)	
	Employed	153 (67.40)	
	Retired	34 (14.98)	
Prior decision-making experience			
	No	197 (86.78)	
	Yes	30 (13.22)	
Decision-making engagement			
	Passive	18 (7.93)	
	Collaborative	193 (85.02)	
	Active	16 (7.05)	

*APACHE II* Acute Physiology and Chronic Health Evaluation II, *M* Mean value, *SD* Standard deviation

Repeated-measures ANOVA indicated that decision regret levels among surrogate decision-makers initially increased and subsequently decreased from T1 to T3, showing a statistically significant change over time (*F* = 28.190, *p* < 0.001). Anxiety (*F* = 461.731, *p* < 0.001) and depressive symptoms (*F* = 250.241, *p* < 0.001) both decreased significantly across the three assessments.

Pearson correlation analyses revealed that decision regret was significantly and positively correlated with both anxiety and depression at all three time points (*p* < 0.001). The correlation matrix for key variables is presented in Table 2.

**Table 2:** Descriptive and correlation statistics for surrogate decision-makers’ decision regret, anxiety and depression symptoms

	M(SD)		F	Decision regret (T1)	Decision regret (T2)	Decision regret (T3)	Anxiety (T1)	Anxiety (T2)	Anxiety (T3)	Depression (T1)	Depression (T2)	Depression (T3)
Decision regret T1	15.21	17.25	461.73***	1								
Decision regret T2	19.20	20.74		0.81***	1							
Decision regret T3	13.58	17.33		0.76***	0.90***	1						
Anxiety T1	12.65	2.67	250.24***	0.27***	0.32***	0.33***	1					
Anxiety T2	10.51	2.78		0.35***	0.49***	0.52***	0.78***	1				
Anxiety T3	8.90	2.68		0.35***	0.48***	0.51***	0.72***	0.85***	1			
Depression T1	11.03	2.75	28.19***	0.31***	0.34***	0.37***	0.86***	0.70***	0.67***	1		
Depression T2	9.39	3.22		0.38***	0.50***	0.52***	0.76***	0.87***	0.84***	0.77***	1	
Depression T3	7.80	2.96		0.43***	0.54***	0.59***	0.67***	0.80***	0.88***	0.66***	0.85***	1

Decision regret is presented as a continuous variable. The left side of the table shows means and standard deviations for each variable at each assessment time point. Repeated‑measures ANOVA results are also provided for each variable. The right section presents the results of correlation analyses among the variables. T1, 1 month after the onset of impaired consciousness; T2, 3 months after onset; T3, 6 months after onset. ***p < 0.001

### Cross-lagged panel analysis

**Fig. 3:**
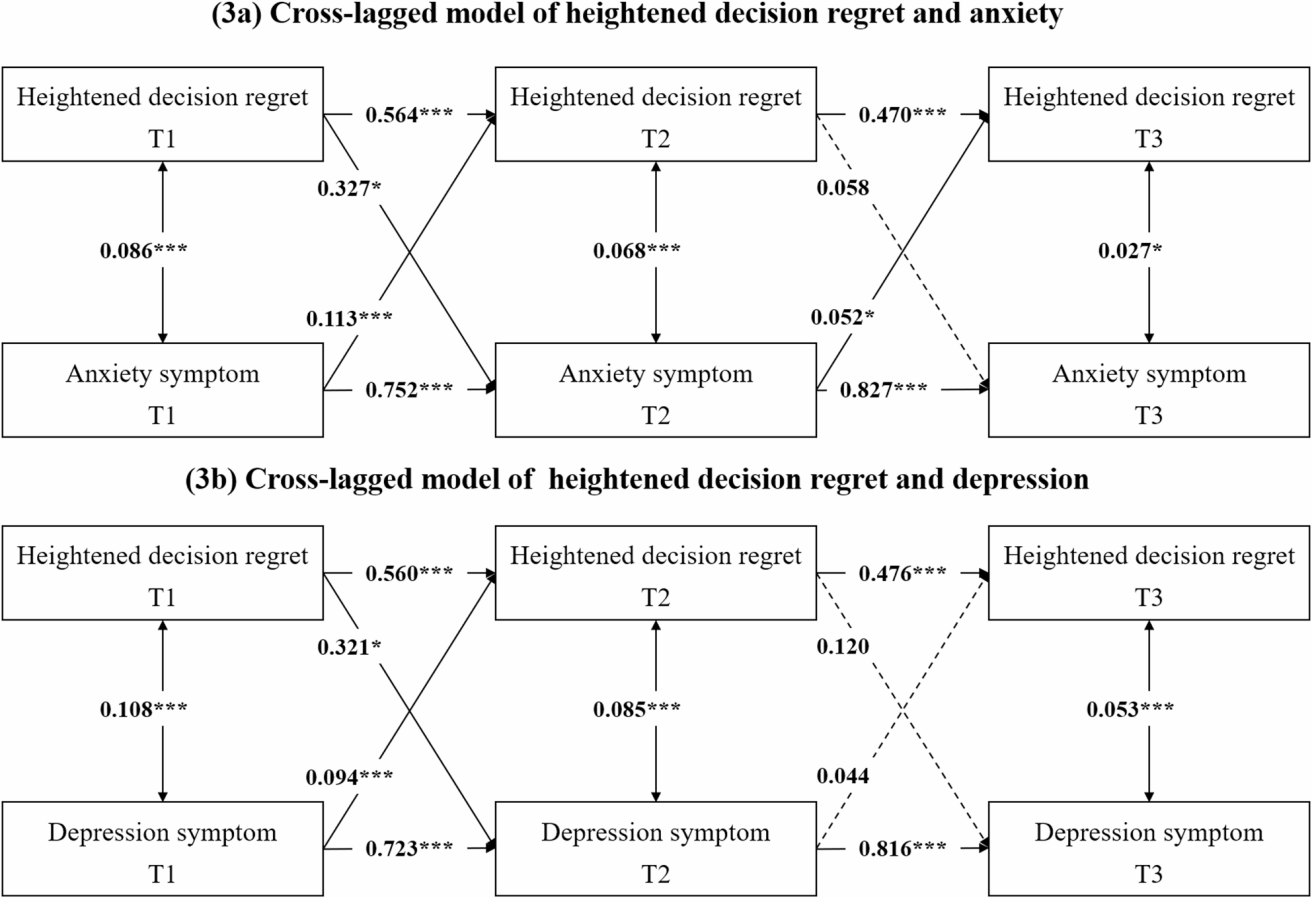
The cross-lagged panel model of heightened decision regret and anxiety (3a), depression (3b). Note: Heightened decision regret was defined as a dichotomous variable indicating a DRS score greater than 25 points. T1, 1 month after the onset of impaired consciousness; T2, 3 months after onset; T3, 6 months after onset. *p＜0.05, ***p＜0.001

The cross-lagged analysis between heightened decision regret and anxiety is presented in Fig. 3a. The model demonstrated acceptable fit: CFI = 0.972, TLI = 0.902, RMSEA = 0.130, SRMR = 0.031. Although the RMSEA was slightly above 0.08, the CFI, TLI, and SRMR all indicated good fit. Considering the relatively small sample size and low degrees of freedom in this study, the RMSEA tends to be overestimated, and its 90% confidence interval (0.076, 0.190) includes 0.08. Overall, the model fit was judged as acceptable. Furthermore, we only tested the prespecified full cross‑lagged model without performing model modifications, in order to fully explore the temporal relationships among the variables. Analysis of cross-lagged paths indicated that heightened decision regret at T1 significantly and positively predicted anxiety symptoms at T2 (*β* = 0.327, *p* < 0.05), whereas heightened decision regret at T2 did not significantly predict anxiety at T3 (*β* = 0.058, *p* > 0.05). Conversely, anxiety at T1 significantly predicted heightened decision regret at T2 (*β* = 0.113, *p* < 0.001), and anxiety at T2 also significantly predicted heightened decision regret at T3 (*β* = 0.052, *p* < 0.05).

The cross-lagged analysis between heightened decision regret and depression is shown in Fig. 3b. This model also demonstrated acceptable fit: CFI = 0.990, TLI = 0.965, RMSEA = 0.079, SRMR = 0.026. Specifically, heightened decision regret at T1 significantly and positively predicted depressive symptoms at T2 (*β* = 0.321, *p* < 0.05), whereas heightened decision regret at T2 did not significantly predict depressive symptoms at T3 (*β* = 0.120, *p* > 0.05). In the reverse direction, depressive symptoms at T1 significantly predicted heightened decision regret at T2 (*β* = 0.094, *p* < 0.05), but depressive symptoms at T2 did not significantly predict heightened decision regret at T3 (*β* = 0.044, *p* > 0.05).

## Discussion

This study investigated the temporal relationship between decision regret and symptoms of anxiety and depression among surrogate decision‑makers of neurocritical patients with pDoC. The results showed that from T1 to T2, heightened decision regret had bidirectional relationships with anxiety and with depression, respectively; from T2 to T3, only anxiety symptoms predicted heightened decision regret. While the cross-lagged panel model supports the temporal sequence among the variables, the study design cannot exclude the potential for unmeasured confounders or reverse causation. Accordingly, the observed relationships should be understood as temporal associations rather than definitive causal mechanisms.

Consistent with our findings, Wen and colleagues also reported a trajectory in which decision regret among surrogate decision-makers for cancer patients initially increased and then gradually decreased from the time of end-of-life decision-making through two years post-bereavement [24]. In our study, decision regret among surrogates for neurocritical patients with pDoC similarly showed an initial increase followed by a subsequent decline. During the early stage of hospitalization, surrogates face numerous clinical decisions, most of which must be made under significant time constraints, thereby increasing their vulnerability to experiencing regret. As treatment progresses and the patient’s condition stabilizes into a pDoC, surrogate decision-makers gradually adapt to and accept this clinical reality. This psychological adaptation process likely contributes to the observed decline in decision-related regret over time. According to cognitive dissonance theory [25], individuals tend to rationalize irrevocable decisions by adjusting their cognitive appraisal, thereby constructing a self-consistent narrative that presents the choice as optimal under the circumstances. This cognitive realignment may help alleviate the surrogate’s sense of regret as they reconcile with the outcome.

Furthermore, although symptoms of anxiety and depression among surrogate decision-makers generally decrease over time, their prevalence remains consistently elevated compared to the general population [14]. This persistent emotional burden not only adversely affects the mental health of surrogates but may also diminish their overall quality of life [26]. Therefore, healthcare professionals should continuously evaluate surrogates’ emotional status during clinical interactions and remain vigilant in identifying psychological distress, including decision regret, anxiety, and depression.

Cross-lagged analysis revealed a bidirectional relationship over time between heightened decision regret and symptoms of anxiety and depression from T1 to T2. Specifically, heightened decision regret at T1 exhibited a significant positive correlation with anxiety and depression at T2, and anxiety and depression at T1 exhibited a significant positive correlation with heightened decision regret at T2, which aligns with previous findings. Multiple studies have shown that anxiety and depression are risk factors for decision regret among surrogate decision-makers [6, 15, 27]. Uncertain patient prognosis, complex and urgent medical decisions, and heavy financial burden predispose surrogate decision-makers to anxiety and depression [13, 14]. These emotions significantly increase cognitive load, impair the processing and integration of complex medical information, and hinder accurate understanding of treatment options. They also cause surrogate decision-makers to persistently worry that decision biases may lead to adverse outcomes or expectation discrepancies. Consequently, decision avoidance and counterfactual thinking emerge, resulting in heightened decision regret [28]. Moreover, heightened decision regret increases the risk of developing anxiety and depression in surrogate decision-makers. Those experiencing heightened decision regret often engage in persistent assumptions about unchosen options. This pattern of upward counterfactual thinking functions as an intrusive cognitive process that amplifies anxiety symptoms by imposing additional psychological burden. Furthermore, when decisions lead to adverse outcomes, intense self-blame through inward attribution may arise. This often triggers feelings of helplessness and meaninglessness, alongside negative perceptions of the self, world, and future [7, 29]. This cascade of negative cognitive-emotional responses ultimately manifests as increased depressive symptoms [20, 30]. These findings support the theory of ambiguous loss [10], in which the uncertainty surrounding the disease status and prognosis of pDoc patients triggers a mutual influence between anxiety and depression and decision regret among surrogate decision-makers.

Another finding was that the bidirectional relationship between heightened decision regret and anxiety and depression disappeared during the T2–T3 period. The disappearance of this bidirectional relationship does not imply that decision regret is no longer important; rather, it may reflect that surrogate decision-makers have partially mitigated the impact of decision regret on anxiety and depression through adaptation, social support, or meaning-making. Additionally, in the T2–T3 phase, only T2 anxiety symptoms predicted heightened decision regret at T3, whereas depression did not. This asymmetry may stem from the distinct characteristics of anxiety and depression [31]. The core feature of anxiety involves worry about future uncertainty and rumination over past decisions [32]. Persistent rumination about patient prognosis and doubt about prior decisions can continuously generate regret. In contrast, the core features of depression are hopelessness and anhedonia. In the later stage of illness, when the patient’s condition is stable but without significant improvement, depression may manifest more as a persistent, relatively static emotional state, with a weakened association with specific decision‑related cognitive activities. Consequently, depression no longer predicts heightened decision regret.

The findings of this study suggest that the bidirectional relationship between heightened decision regret and anxiety and depression may occur primarily in the early stage of illness (1–3 months); therefore, this period may be a critical window for early intervention. After three months, psychological problems amongst surrogate decision-makers may stem more from chronic burden than from ongoing decision regret, and the focus of intervention may be best shifted toward general emotional regulation and stress management. Therefore, future clinical practice might implement a staged intervention protocol for surrogate decision-makers based on the patient’s disease course. In the early stage of illness (1–3 months), psychological support including regular family meetings might focus on alleviating decisional conflict among surrogate decision-makers, thereby interrupting the mutual reinforcement with anxiety and depression [33]. In the later stage of illness (3–6 months), only anxiety symptoms predict decision regret, suggesting that intervention during this period should likely primarily focus on identifying and managing anxiety. Approaches such as writing ICU diaries [34], involving surrogate decision‑makers in patient care, implementing flexible visiting policies [35], and applying cognitive behavioral therapy [36] can help surrogate decision‑makers understand patient prognosis, reduce uncertainty, decrease counterfactual thinking, and thereby alleviate anxiety and mitigate decision regret.

### Strengths and limitations

The main strength of this study lies in its prospective longitudinal design, which captures the dynamic changes in decision regret and anxiety and depressive symptoms over time, and the use of cross-lagged analysis to clarify a bidirectional relationship between heightened decision regret and symptoms of anxiety and depression.

However, several limitations should be noted. First, this study employed convenience sampling, and the sample was drawn from only two tertiary hospitals in the same region, resulting in a relatively small sample size. Future multi-center studies with larger samples are warranted.

Second, this study used a GCS score of < 12 as the clinical screening criterion for patient enrollment. However, the GCS has certain limitations in assessing consciousness in pDoC patients. The absence of the more standardized Coma Recovery Scale–Revised (CRS-R) may have introduced heterogeneity in the diagnosis of disorders of consciousness, which is a limitation of this study. Notably, standardized tools involving caregivers in consciousness assessment have recently emerged, such as the Social and Family Evaluation (SAFE) scale developed by Magnani et al. [37]. Future studies may incorporate the SAFE scale to both enrich diagnostic information and demonstrate attention to the experiences of surrogate decision‑makers.

Third, excluding surrogate decision‑makers of deceased patients during follow‑up potentially overestimating the levels of decision regret, anxiety, and depression and the strength of bidirectional relationships. Patient death terminates ambiguous loss, potentially reducing anxiety, depression, and decision regret. Future research should apply an intention‑to‑follow‑up approach and analyze surrogate decision‑makers of deceased patients to test the robustness of our findings.

Fourth, the hospitals where the study was conducted included multiple ICUs. Anoxic brain injury was primarily admitted to the emergency ICU, whereas the neurosurgical and neurological ICUs mainly admitted patients with traumatic brain injury, cerebrovascular disease, brain tumors, and so on. The study sample only included the pDoC etiologies actually admitted to the neurosurgical and neurological ICUs, and did not include anoxic brain injury. The latter group may face poorer prognoses and decisions about withdrawal of life-sustaining treatment, and the psychological trajectories of their surrogates might differ from our findings. Future research could compare the psychological trajectories of surrogate decision‑makers across different etiologies and explore the unique impact of WLST decision contexts on their psychological outcomes.

Fifth, this study focused on the psychological variables of surrogate decision‑makers themselves. Due to limitations in sample size, potential covariates such as patient disease type and clinical course, the relationship between surrogate decision‑maker and patient, and prior decision‑making experience could not be included in the cross‑lagged model. Future studies should expand the sample size and incorporate these variables as control variables in the model.

Finally, the follow-up period was limited to six months due to practical constraints. Given the extended recovery trajectory typical of neurocritical illnesses, future research should incorporate longer follow-up durations to more comprehensively investigate the longitudinal relationships between decision regret and symptoms of anxiety and depression among surrogate decision-makers throughout the treatment and recovery process.

## Conclusions

This study investigated the bidirectional relationships between decision regret and symptoms of anxiety and depression in surrogate decision-makers of neurocritical patients with pDoC. The findings indicate that decision regret among surrogates peaked and subsequently declined within six months after the patient’s diagnosis, while anxiety and depressive symptoms gradually decreased over the same period. Furthermore, a bidirectional relationship was identified between heightened decision regret and anxiety and depression. Therefore, healthcare professionals should closely monitor the psychological state of these surrogates and implement timely, evidence-based interventions to mitigate decision regret and co-occurring anxiety and depression.

## Data Availability

The datasets generated during this study are available from the corresponding author upon reasonable request.​.

## Abbreviations

pDoC: Prolonged Disorders of Consciousness
ICU: Intensive Care Unit
DRS: Decision Regret Scale
HADS: Hospital Anxiety and Depression Scale
APACHE II: Acute Physiology and Chronic Health Evaluation II
M: Mean value
SD: Standard deviation
CFI: Comparative Fit Index
TLI: Tucker-Lewis Index
RMSEA: Root Mean Square Error of Approximation
SRMR: Standardized Root Mean Square Residual
WLST: Withdrawal of Life-Sustaining Treatment
